# Race, Ethnicity, and Language Disparities in Alcohol and Drug Screening and Medication Treatment

**DOI:** 10.1001/jamanetworkopen.2026.12319

**Published:** 2026-05-13

**Authors:** Brian Chan, David Ezekiel-Herrera, Steffani R. Bailey, Elena Byhoff, Miguel Marino, Jennifer A. Lucas, Sophia Giebultowicz, Kevin Espinoza, Zoe Larson, John Heintzman

**Affiliations:** 1 Division of General Internal Medicine and Geriatrics, Addiction Medicine Section, Oregon Health & Science University, Portland; 2 Department of Family Medicine, Oregon Health & Science University, Portland; 3 Division of Biostatistics, School of Public Health, Oregon Health & Science University, Portland; 4 OCHIN Inc, Portland, Oregon; 5 Health Choice Network, Miami, Florida; 6 Central City Concern, Portland, Oregon; 7 Department of Medicine, UMass Chan Medical School, Worcester, Massachusetts

## Abstract

**Background:**

Unhealthy alcohol and drug use have significant health-related sequelae. Given racial and ethnic disparities in complications of substance use, successful screening and medication prescribing for addictions are important in community health settings serving diverse populations.

**Objective:**

To evaluate alcohol and drug use screening and prescribing of medications for addiction treatment in adults by race, ethnicity, and language preference.

**Design, Setting, and Participants:**

This cohort study included US adults seen between 2012 and 2020 in a multistate electronic health record (EHR) network (1394 primary care clinics). Analyses were completed October 2024.

**Exposure:**

Race and ethnicity with language preference groups: non-Hispanic White, non-Hispanic Black, Latino with Spanish language preferred, and Latino with English language preferred.

**Main Outcome(s) and Measures:**

Multivariable logistic regression estimated covariate-adjusted odds ratios (aOR) of receipt of alcohol and drug screening and EHR-documented prescription of medication for alcohol (AUD) or opioid use disorders (OUD).

**Results:**

There were 2 191 945 patients across 25 states (mean (SD) age, 41.3 [15.2] years; 1 236 818 female [56.4%]); 416 607 identified as non-Hispanic Black (19.0%), 1 015 066 non-Hispanic White (46.3%), 474 389 Latino with Spanish-language preference (21.6%), and 285 883 Latino with English-language preference (13.0%). Over the study period, 869 609 (39.7%) had documented completed alcohol screening, and 862 263 (39.3%) completed drug screening—113 629 (5.2%) had a diagnosis of AUD and 247 530 (11.3%) had an OUD diagnosis. Spanish-preferring Latino patients had 59% increased odds of screening compared with non-Hispanic White patients (aOR, 1.59; 95% CI, 1.31-1.93). All minoritized race and ethnicity with language preference groups had lower odds of prescribed medications for addictions treatment compared with non-Hispanic White patients; non-Hispanic Black patients had the lowest odds of any group (AUD: aOR, 0.55; 95% CI, 0.43-0.69; OUD: aOR, 0.38; 95% CI, 0.31-0.46).

**Conclusions and Relevance:**

In this cohort study, there was an overall low likelihood of completed screening for alcohol and drug use among all minoritized race and ethnicity with language preference groups. All minoritized groups had lower odds of receipt of medications for addiction treatment compared with the non-Hispanic White group. Improving screening and addressing this emerging treatment inequity should be prioritized.

## Key Points

**Question:**

In a nationally representative network of community health centers serving diverse patients, are there differences in evidence-based alcohol and drug screening and prescribing for addiction treatment by race, ethnicity, and language preference?

**Findings:**

In this cohort study of health records for 2 million US adults across 1300 clinics, 40% ever had alcohol and drug screening completed. While Latino patients preferring Spanish were most likely to have received screening, all patients from minoritized racial and ethnic groups (including Latino patients with both Spanish or English language preference) had significantly lower odds of prescribed medications for addiction compared with non-Hispanic White patients.

**Meaning:**

These results suggest that increasing substance use screening and addressing the emerging treatment inequity should be prioritized.

## Teaser

This cohort study of US adults examines differences in rates of alcohol and drug screening and prescribing for addiction by race and Hispanic ethnicity by language preference.

## Introduction

The Centers for Disease Control and Prevention characterizes excessive alcohol use as a leading preventable cause of death in the US, as well as leading to chronic diseases and serious health problems including high blood pressure, heart disease, liver disease, and cancer.^1^ Unhealthy alcohol use (which includes use above recommended limits, use that adversely affects health, or alcohol use disorder [AUD])^2^ was exacerbated by the COVID-19 pandemic; one study found that the observed AUD mortality rates increased by 24.8% in 2020 and 21.95% in 2021.^3^ The 2021 National Survey on Drug Use and Health found that 13.5% of people ages 12 years and older who drink alcohol reported drinking more than they did before the pandemic began.^4^ Similarly, rates of opioid use disorder (OUD) incidence and opioid overdose deaths have increased from 2002 until 2022, driven by the rise of synthetic opioids (ie, fentanyl).^5,6^

Screening and brief interventions in primary care, such as counseling and treatment initiation and referral (SBIRT [screening, brief intervention, and referral to treatment]), may help circumvent adverse health outcomes associated with drinking. Screening has been shown to reduce alcohol use and improve mental and physical health,^7,8^ as well as halt the development of more severe alcohol consumption.^2^ While there is less robust evidence for screening for nonalcohol related substances^9,10,11,12^ similar approaches of screening, treatment initiation, and referral (STIR) show promise.^13^ In 2018 the US Preventive Services Task Force (USPSTF) recommended screening for unhealthy alcohol use in primary care settings for adults aged 18 years or older, and, in 2020, recommended similar screening for unhealthy drug use.^2^ Despite this recommendation, screening rates have been variable, with studies identifying screening recommendation adherence as high as 81% to 87%^14,15^ and as low as 2.6%.^16^ Even when alcohol screening rates were high, rates of treatment referral and receipt among those with AUD were much lower, with only 4.3% to 15.5% being referred to treatment, depending on severity, and only 5.8% receiving treatment.^15^

Previous research has shown Latino populations’ drinking patterns differ from non-Hispanic White and other racial groups.^8,17^ However, recent trends show increasing rise in unhealthy alcohol and drug use in Latino and non-Hispanic Black populations, potentially worsening known health disparities.^18^ There are notable differences in prevalence of substance use disorders by language preference, with Latino patients who prefer speaking Spanish less likely to develop alcohol or drug problems compared with English language–preferred counterparts.^19^ There is conflicting evidence on racial and ethnic disparities and screening for alcohol and other drug use,^8,14^ with a 2022 study^20^ showing that minoritized groups receive less screening than White individuals. Regarding treatment for alcohol and other drug use, Latino and Black patients with substance use disorders (SUDs) are less likely to receive treatment compared with White patients,^21^ but data on differences in receipt of medications for addiction treatment is sparse. A Veterans Affairs study^22^ showed that Black patients were less likely to receive medications for alcohol use, though Latino patients had similar rates compared with White patients. For medications for opioid use disorder (MOUD), a large Medicare claims study^23^ found both Latino and Black patients were less likely to receive MOUD; and analysis of commercial insurance claims reveals a trend of decreasing treatment durations compared with White patients.^24^ Cultural and language barriers may be contributing to lower screening and treatment for substance use, as has been shown in mental health.^25,26,27^ No studies have characterized language preference disparities in substance use screening and treatment, an important research gap.^28^

Because community health centers (CHC) serve a disproportionate number of minority patients nationwide of all age ranges and insurance types (including the uninsured), they are important settings for understanding patterns of alcohol and other drug screening and medication initiation interventions.^29^ To address the existing gaps in research on racial and ethnic disparities on screening for alcohol or opioid use disorder and access to medications for addictions, we used a linked electronic health record (EHR) dataset to examine racial and ethnicity with language preference patterns of substance use screening, diagnoses of AUD and OUD in our setting, odds of having medications for addiction treatment prescribed among adult patients seeking care at community health centers from 2012 to 2020.

## Methods

### Population

This is a retrospective cohort study of Latino, non-Hispanic Black, and non-Hispanic White adults who were examined at CHCs between January 1, 2012, to December 31, 2020, in the OCHIN clinical data research network in 25 states. OCHIN (OCHIN Inc) is a health care practice–based research network providing a single instance of the EPIC EHR to safety net clinics, including federally qualified health centers (FQHCs), rural health clinics, school-based health centers, local health departments, and other community-based health centers that deliver care to people who are public insured, uninsured, or otherwise medically underserved.^30^ We defined the population as patients with at least 1 documented primary care visit at a CHC in the study period who were age 18 years or older at their first visit during the study period, and thus eligible for annual screening. Data were collected during routine clinical care and were not specifically collected for these analyses.

This study was approved by the institutional review board of Oregon Health & Science University. Written consent from patients to use data in research was obtained from clinics when care was initiated. We followed the Strengthening the Reporting of Observational Studies in Epidemiology (STROBE) reporting guideline for reporting cohort studies.

### Race and Ethnicity With Language Preference

Our independent variable of interest was a combination of race, ethnicity, and language preference that resulted in 4 mutually exclusive groups: non-Hispanic White, non-Hispanic Black, Latino Spanish-language preferred, Latino English-language preferred. Race, ethnicity, and language preference were based on patient self-reported clinic registration data. We use the term Latino because it is often preferred in our study population; the actual ethnicity variable in the EHR is Hispanic and non-Hispanic. We focus on English-preferring and Spanish-preferring Latino patients because previous research in our network has shown that these groups utilize care differently.^31,32^

### Primary Outcome

Primary outcome was any instance of documented alcohol and drug screening. We defined screening as any EHR-documented entry of an annual questionnaire which contains 2 questions: (1) “Men: Alcohol: How many times in the past year have you had 5 or more drinks in a day (none vs 1 or more); Women: How many times in the past year have you had 4 or more drinks in a day?” (none vs 1 or more); (2) “Drugs: How many times in the past year have you used a recreational drug or used a prescription medication for nonmedical reasons? (none vs 1 or more).” If the screen was positive for the respective substance, additional questionnaires were recorded (eg, alcohol use disorders identification test [AUDIT], Drug Abuse Screening Test [DAST]^33^).

### Secondary Outcomes

#### Diagnosis of Alcohol Use Disorder and Prescription of Medications for Alcohol Use Disorder

We defined a patient as having an AUD diagnosis if they had a recorded AUDIT score above 14 at any point after first screening completed.^34^ We defined prescribed medication for AUD as having at least 1 prescription for any of the following medications that are US Food and Drug Administration (FDA)-approved for AUD including: naltrexone (by mouth or long-acting), disulfiram, or acamprosate.

#### Diagnosis of Opioid Use Disorder and Prescription Medications for Opioid Use Disorder

Because the DAST is not exclusive to opioid use disorder, diagnosis of OUD was defined as a record of an *International Classification of Diseases, Ninth Revision (ICD-9)* or *International Statistical Classification of Diseases and Related Health Problems, Tenth Revision (ICD-10)* OUD diagnosis in the medical record at any point after first screening completed. We defined prescribed medication for OUD as having at least 1 prescription for any FDA-approved medication for OUD that can be prescribed in primary care including naltrexone (by mouth or long-acting) or buprenorphine product (sublingual or long-acting injectable).

#### Covariates

We adjusted for the following potential confounders: age at first encounter, sex, insurance status at visits during the study period (all public, all private, public and private, no insurance), income as a percentage of the federal poverty level (using a cut-off of 138%, which is the common threshold for Medicaid eligibility), number of primary care visits during the study period (as a proxy for health care utilization in general), depression *ICD-9*/*ICD-10* code diagnosis present, and Charlson score as a measure of medical complexity. We also accounted for whether the state ever expanded their Medicaid program after the Affordable Care Act to adjust for state-level heterogeneity.

### Statistical Analysis

We conducted descriptive analyses of patient characteristics for the sample as a whole and by our race and ethnicity with language preference groups. We estimated relative odds of receipt of screening, AUD diagnosis, and among those that had an AUD medical record diagnosis, whether they had been prescribed medications for AUD. We repeated the procedures for OUD. We used multivariable logistic regression with robust standard error estimators clustering on clinic to estimate covariate-adjusted odds of screening between race and ethnicity with language preference groups for all outcomes. For all regression analyses, White patients were considered referent group, and robust standard errors were estimated to account for clustering of patients within clinics. We accounted for clustering at the clinic level to account for within-clinic variability. Analyses were completed October 2024 using Stata version 15 (StataCorp LLC) and R version 4.5.1 (R Project for Statistical Computing) with 2-sided testing and type I error set at 5%; *P* < .05 was used as the threshold for significance.

## Results

### Characteristics of the OCHIN Network Cohort

Between January 1, 2012, and March 20, 2020, there were 2 191 945 patients in the sample across 1394 clinics in 25 states with mean (SD) age of 41.3 (15.2) years at first visit. The majority of patients were of female sex (1 236 818 [56.4%]); 491 356 (22.4%) were never insured, while 1 138 963 (51.9%) reported some public insurance. The cohort had moderate medical complexity with 40% having Charlson scores 2 or higher. Few patients (202 332 [9.2%]) had documented current or history of homelessness and 455 512 (20.8%) had an *ICD-9* or *ICD-10* diagnosis of depression. There were 416 607 non-Hispanic Black (19.0%), 1 015 066 non-Hispanic White (46.3%), 474 389 Latino with Spanish-language preferred (21.6%), and 285 883 Latino with English-language preferred (13.0%) patients. The cohort had 14% residing in Medicaid nonexpansion states—there were disproportionately more Black patients residing in nonexpansion states (141 612 [34.0%]) compared with Latino (90 551 [11.9%]) and White (78 549 [7.7%]) patients (Table).

**Table.** Description of the Patient Sample by Race, Ethnicity, and Language Preference

Characteristic	Patients, No. (%)				
	Overall (n = 2 191 945)	Race and ethnicity with language preference groups			
		Latino preferring English (n = 285 883)	Latino preferring Spanish (n = 474 389)	Non-Hispanic Black (n = 416 607)	Non-Hispanic White (n = 1 015 066)
Age at first encounter, mean (SD), y	41.3 (15.2)	33.9 (13.3)	42.7 (14.0)	40.4 (15.0)	43.1 (15.8)
Age group, y					
18-39	1 101 948 (50.3)	204 241 (71.4)	211 358 (44.6)	215 383 (51.7)	470 966 (46.4)
40-49	393 677 (18)	37 994 (13.3)	115 458 (24.3)	73 403 (17.6)	166 822 (16.4)
50-79	696 320 (31.8)	43 648 (15.3)	147 573 (31.1)	127 821 (30.7)	377 278 (37.2)
Sex					
Male	955 127 (43.6)	117 347 (41.0)	180 772 (38.1)	193 151 (46.4)	463 857 (45.7)
Female	1 236 818 (56.4)	168 536 (59.0)	293 617 (61.9)	223 456 (53.6)	551 209 (54.3)
Visits, No./y					
<1	741 412 (33.8)	93 830 (32.8)	126 430 (26.7)	156 714 (37.6)	364 438 (35.9)
1-3	677 952 (30.9)	89 267 (31.2)	147 572 (31.1)	121 047 (29.1)	320 066 (31.5)
3-5	306 732 (14)	38 394 (13.4)	77 461 (16.3)	55 159 (13.2)	135 718 (13.4)
≥5	465 849 (21.3)	64 392 (22.5)	122 926 (25.9)	83 687 (20.1)	194 844 (19.2)
Insurance					
Never insured	491 356 (22.4)	55 858 (19.5)	145 524 (30.7)	108 915 (26.1)	181 059 (17.8)
Some private	380 308 (17.4)	37 571 (13.1)	62 002 (13.1)	50 290 (12.1)	230 445 (22.7)
Some public and private	181 318 (8.3)	21 003 (7.3)	42 985 (9.1)	32 771 (7.9)	84 559 (8.3)
Some public	1 138 963 (52)	171 451 (60)	223 878 (47.2)	224 631 (53.9)	519 003 (51.1)
FPL					
Sometimes over, sometimes under 138%	204 822 (9.3)	21 844 (7.6)	53 444 (11.3)	26 046 (6.3)	103 488 (10.2)
Always ≥138%	261 570 (11.9)	29 876 (10.5)	36 139 (7.6)	33 577 (8.1)	161 978 (16)
Always <138%	1 219 186 (55.6)	167 118 (58.5)	316 694 (66.8)	283 892 (68.1)	451 482 (44.5)
Never documented	506 367 (23.1)	67 045 (23.5)	68 112 (14.4)	73 092 (17.5)	298 118 (29.4)
Homeless status					
Always homeless	85 115 (3.9)	10 125 (3.5)	8671 (1.8)	31 828 (7.6)	34 491 (3.4)
Ever homeless	117 217 (5.3)	14 902 (5.2)	19 471 (4.1)	30 751 (7.4)	52 093 (5.1)
Never homeless	1 393 223 (63.6)	203 531 (71.2)	344 976 (72.7)	280 015 (67.2)	564 701 (55.6)
No information	596 390 (27.2)	57 325 (20.1)	101 271 (21.3)	74 013 (17.8)	363 781 (35.8)
Foreign born					
US born	126 664 (5.8)	25 554 (8.9)	7041 (1.5)	44 432 (10.7)	49 637 (4.9)
Not US born	87 814 (4)	8284 (2.9)	67 789 (14.3)	8137 (2)	3604 (0.4)
No information	1 977 467 (90.2)	252 045 (88.2)	399 559 (84.2)	364 038 (87.4)	961 825 (94.8)
Charlson Score					
0	936 620 (42.7)	152 878 (53.5)	240 664 (50.7)	172 381 (41.4)	370 697 (36.5)
1	377 569 (17.2)	49 118 (17.2)	94 175 (19.9)	70 988 (17)	163 288 (16.1)
≥2	877 756 (40)	83 887 (29.3)	139 550 (29.4)	173 238 (41.6)	481 081 (47.4)
No recorded pregnancies	2 050 043 (93.5)	255 412 (89.3)	427 707 (90.2)	394 234 (94.6)	972 690 (95.8)
Depression diagnosis	455 512 (20.8)	49 708 (17.4)	66 654 (14.1)	71 641 (17.2)	267 509 (26.4)
Rural	39 676 (1.8)	940 (0.3)	708 (0.1)	611 (0.1)	37 417 (3.7)
Medicaid Expansion State status					
Expanded 2014	1 590 237 (72.5)	226 662 (79.3)	360 136 (75.9)	198 678 (47.7)	804 761 (79.3)
Expanded 2015	67 064 (3.1)	3315 (1.2)	3528 (0.7)	23 824 (5.7)	36 397 (3.6)
Expanded 2016	28 078 (1.3)	1035 (0.4)	210 (0)	840 (0.2)	25 993 (2.6)
Expanded 2019	180 798 (8.2)	24 864 (8.7)	46 180 (9.7)	51 272 (12.3)	58 482 (5.8)
Expanded 2020	15 056 (0.7)	1879 (0.7)	1912 (0.4)	381 (0.1)	10 884 (1.1)
Never expanded	310 712 (14.2)	28 128 (9.8)	62 423 (13.2)	141 612 (34)	78 549 (7.7)
Outcomes					
Any alcohol screening completed	869 609 (39.7)	109 149 (38.2)	213 308 (45)	142 042 (34.1)	405 110 (39.9)
Any drug screening completed	862 263 (39.3)	108 288 (37.9)	211 147 (44.5)	139 503 (33.5)	403 325 (39.7)
Any positive alcohol screens					
Never screened	1 322 336 (60.3)	176 734 (61.8)	261 081 (55)	274 565 (65.9)	609 956 (60.1)
No positive screens	672 749 (30.7)	86 154 (30.1)	185 852 (39.2)	110 090 (26.4)	290 653 (28.6)
≥1 Positive screens	196 860 (9)	22 995 (8)	27 456 (5.8)	31 952 (7.7)	114 457 (11.3)
Any positive drug screens					
Never screened	1 329 682 (60.7)	177 595 (62.1)	263 242 (55.5)	277 104 (66.5)	611 741 (60.3)
No positive screens	735 425 (33.6)	94 452 (33)	207 149 (43.7)	115 816 (27.8)	318 008 (31.3)
≥1 Positive screens	126 838 (5.8)	13 836 (4.8)	3998 (0.8)	23 687 (5.7)	85 317 (8.4)
AUDIT positive	113 629 (5.2)	13 009 (4.6)	13 555 (2.9)	17 715 (4.3)	69 350 (6.8)
OUD diagnosis	247 530 (11.3)	24 860 (8.7)	13 315 (2.8)	52 511 (12.6)	156 844 (15.5)
Any AUD medication prescription^a^	3296 (3.0)	283 (2.7)	253 (2.4)	495 (2.3)	2265 (3.3)
Any OUD medication prescription^b^	18 597 (7.5)	1869 (7.5)	587 (4.4)	1659 (3.2)	14482 (9.2)

Abbreviations: AUD, alcohol use disorder; AUDIT, Alcohol Use Disorders Identification Test; FPL, Federal Poverty Level; OUD, opioid use disorder.

^a^ Among patients with AUD diagnosis.

^b^ Among patients with OUD diagnosis.

### Adjusted Odds of Ever Screened for Alcohol and Drugs

Among the total cohort, 869 609 (39.7%) had completed at least 1 alcohol screening, and 862 263 (39.3%) completed at least 1 drug screening. In the adjusted analysis, only Latino patients who preferred Spanish had increased odds of having a documented alcohol screening compared with White patients (aOR, 1.59; 95% CI, 1.31-1.93) (Figure). Latino patients who preferred English (aOR, 1.07; 95% CI, 0.92-1.25) and Black patients (aOR, 0.87; 95% CI, 0.73-1.04) had similar odds of screening compared with White patients. Odds of drug screening were similar (Figure, A).

**Figure.**
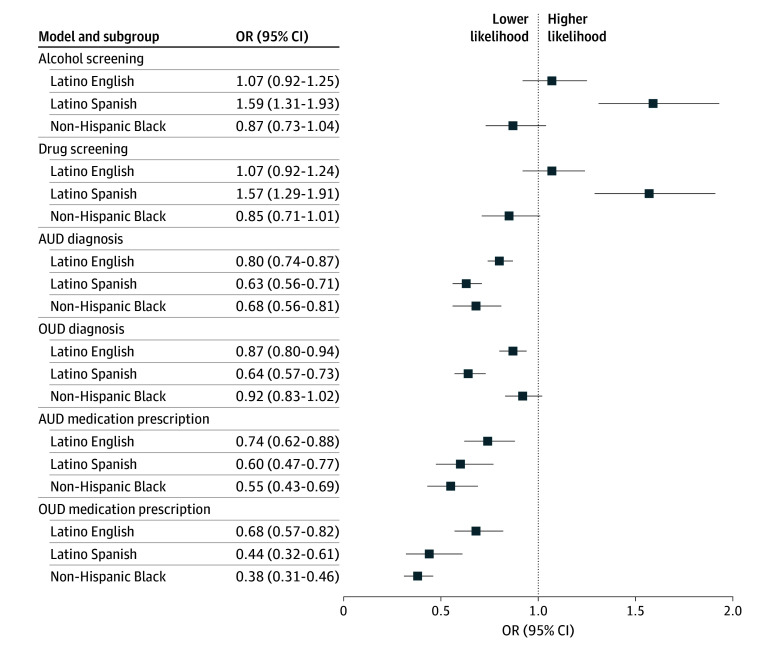
Forest Plot of Odds Ratios (ORs) for Screening for Alcohol Use, Screening for Drug Use, Diagnosis of Alcohol Use Disorder (AUD) and Opioid Use Disorder (OUD), and Prescription Medications for AUD and OUD

### Adjusted Odds of Diagnosis of AUD and OUD

The overall prevalence of AUD diagnosis in the cohort was 5.2% (113 629 patients) (Table). White patients had the highest unadjusted prevalence (69 350 [6.8%]) of AUD diagnosis and Latino patients who preferred Spanish language had the lowest (13 555 [2.9%]). After covariate adjustment, all race and ethnicity with language preference groups had decreased odds of AUD diagnosis compared with NHW. Compared with White patients, Latino patients who preferred Spanish language had 36% reduced odds of having an AUD diagnosis (aOR, 0.63; 95% CI, 0.56-0.71), Black patients had 32% reduced odds (aOR, 0.68; 95% CI, 0.56-0.81), and Latino patients who preferred English language patients had 20% reduced odds of an AUD diagnosis (aOR, 0.80; 95% CI, 0.74-0.87).

Similarly, unadjusted prevalence of OUD differed by race and ethnicity with language preference, with White patients having highest prevalence compared with all other groups. In the covariate adjusted analysis, both Latino patients who preferred Spanish (aOR, 0.64; 95% CI, 0.57-0.73) and Latino patients who preferred English (aOR, 0.87; 95% CI, 0.80-0.94) had lower odds of having an OUD compared with White patients; Black patients had similar odds of an OUD (aOR, 0.92; 95% CI, 0.83-1.02).

### Adjusted Odds of Medications for AUD and OUD

We also found low prevalence (3296 [3.0%]) of documented prescription orders for FDA-approved medications for AUD among those with an AUD diagnosis. After adjustment, similar to AUD diagnosis, all race and ethnicity with language preference groups had markedly lower odds of documented prescription for medications for AUD as compared with White patients (Figure, A). Black patients with AUD had 45% reduced odds of having a documented prescription for medication for AUD compared with White patients (aOR, 0.55; 95% CI, 0.43-0.69). Latino patients who preferred Spanish had reduced odds of being prescribed medication treatment for AUD (aOR, 0.60; 95% CI, 0.47-0.77), followed by Latino patients who preferred English (aOR, 0.74; 95% CI, 0.62-0.88), compared with White patients.

The prevalence of documented prescribed MOUD was higher (18 597 [7.5%]) than for medications for AUD, but still low, and there were greater disparities in prescribing MOUD across groups. In the adjusted analysis, we found that Black patients had the lowest odds of documented MOUD (aOR, 0.38; 95% CI, 0.31-0.46), followed by Latino patients who preferred Spanish language (aOR, 0.44; 95% CI, 0.32-0.61) and Latino patients who preferred English (aOR, 0.68; 95% CI, 0.57-0.82).

## Discussion

In this study of a racially and ethnically diverse population seen in a multistate network of community-based primary care clinics between 2012-2020, we observed overall low likelihood of completed screening for alcohol and drug use. Among those screened, only the Latino Spanish-preferred group had higher odds of screening compared with White patients; all other race and ethnicity with language preference groups had similar odds of screening. For AUD and OUD diagnoses and medications for addictions treatment prescriptions, we found that all minoritized groups were less likely to be diagnosed with an AUD or OUD, and among those with an AUD or OUD, lower odds of EHR-documented medication prescriptions for addiction treatment. The overall low screening and low prescribing of medications for addiction treatment suggests more work to be done to achieve equitable substance use care in community primary care.

Unhealthy alcohol and drug use remain a significant contributors to morbidity and mortality, associated with 178 000 (alcohol)^35^ and 81 000 (opioids) deaths in the US annually, at great societal costs.^36^ Despite evidence based interventions for screening and intervention, and the USPSTF recommendation to routinely screen for alcohol and drug use in primary care,^37^ uptake of this practice in primary care remains low and uncommon.^16^ This study, the first to report alcohol and drug screening in a large community health center network, confirms this gap, with 60% having never had a documented screening across the 8-year period. Other studies of alcohol screening in primary care using survey data from the Behavioral Risk Factor Surveillance Survey and National Survey on Drug Use and Health have found similar rates of unhealthy alcohol screening.^14,38,39^ While there is lower quality evidence to support routine screening for drug use in primary care, the USPSTF did recommend this practice in 2020, and we saw similar likelihood of drug screening as for alcohol screening. This may reflect how alcohol and drug screening is implemented in practice, often administered together by patient self-report with follow-up by clinicians based on the results.

While overall implementation of comprehensive alcohol and drug screening can be improved, the finding of increased (for Latino patients who prefer Spanish) or similar (for Latino patients who prefer English and Black patients) odds of screening for both alcohol and drug use when compared with White patients in our sample is encouraging. Studies to date have shown Black and Latino people have lower odds of developing lifetime AUD^40,41^ and OUD compared with White patients.^42^ But recent trends indicate disproportionate increases in SUD rates in these groups.^43,44^ Thus, increased attention to ensure all patients receive evidenced-based screening for SUDs is important. While other screening studies (eg, cancer,^31,45^ hepatitis C^32^ screening) in our network have shown similar pattern of minority populations having higher odds of screening, our finding contrasts with other studies using nationally representative surveys that report non-White persons are less likely to be screened for unhealthy alcohol use.^46^ Our data were from community clinics that receive federal funds to tailor care for their populations, and this finding may be due to increased attention by these clinics and health care professionals to address the health needs of the diverse populations they serve, such as utilizing language concordant services and by employing people who live in the communities being served.^47,48^

Also concerning were the overall low rates of patients in our network who were prescribed medications for either alcohol or opioid use disorders. Our reported prescribing rates of medications for AUD are similar to what is reported nationally,^49^ but considerably lower for OUD (eg, buprenorphine).^50^ A 2024 study^27^ of mental health utilization in our network that reported buprenorphine prescribing also found similar low rates of documented prescriptions for patients with OUD. Improving uptake of medications for addiction treatment in primary care is needed, and to ensure this is done equitably given minoritized populations with SUDs often have more disease severity, higher rates of complications from SUDs (eg, liver disease, overdose), and less uptake and engagement with treatment, including use of medications for addiction treatment.^40,51,52^ Unfortunately, we did find disparities in recorded prescriptions for medications for addiction treatment. Even adjusting for insurance and Medicaid expansion status, Black and Latino patients had lower odds of receipt of evidenced based medications for both AUD and OUD. This pharmacoinequity^53^ is concerning though not surprising, as seen in other studies using commercial claims data.^23^ The reasons for pharmacoinequity are multifactorial—access and cost barriers to medications, prescribing behaviors of physicians, reluctance of individuals to receive treatment due to mistrust or lack of familiarity with treatment, and other social determinants—especially around racism and incarceration—may exacerbate this.^54,55^ More work can be done in our network of CHCs to understand which determinants are at play.

### Differences by Preferred Language and Potential Role of Acculturation

We found Latino patients who preferred Spanish had higher odds of receiving SUD screening than Latino patients who preferred English. This finding may reflect how language preference is a marker for acculturation^56^—Latino patients with less acculturation may be more willing to engage in recommended screening interventions than those who are more acculturated. This has been shown in other health outcomes,^57^ although the relationship between acculturation and uptake of preventative health services is not definitive.^58^ What is clearer in prior research is the association of higher acculturation and risk of substance use,^56,58,59,60^ and this may explain our findings of why Latino patients who prefer Spanish had lower odds of having either an AUD or an OUD compared with Latino patients who prefer English and White patients. Acculturation itself is a complex phenomenon that likely intersects with other social determinants of health (eg, racism, socioeconomic position, and other social determinants of health),^61^ so differences between preferred language groups warrant further inquiry.

### Limitations

There are limitations to our study. This is a cohort of patients seen in community-based primary care clinics, which serve a high percentage of minoritized populations, and may not be generalizable to other primary care populations. The OCHIN network is the largest network of community health clinics in the US but may not be nationally representative of certain populations, such as rural patients. We defined whether alcohol and drug screening occurred by documentation in the electronic health record. Discrepancies between patient survey reported completion of screening and documented screening are common and have been reported in multiple samples (eg, screening as informal conversations to speed the visit and reduce patient discomfort).^62,63^ The discrepancy appears to be greater in areas of higher social deprivation.^64^ Our network includes clinics in high-deprivation areas, which could be at risk for lower documented screening. Misclassification bias may be present as medications ordered in the EHR may not be subsequently filled or taken by the patient. We also do not know if medications were offered and the patient refused or whether providers never offered them at all, which would inform future corrective interventions. Similarly, this EHR dataset does not capture use of methadone (a restricted medication dispensed only from a specialty treatment program) as a medication for OUD. It is known that the racial and ethnic composition of a community is associated with which MOUD residents have access to, with methadone more common in highly segregated Latino and Black communities, which may explain the overall low rates and disparities in MOUD prescribing observed.^65^ It is possible that some of the clinics in this network are in areas that did not have access to buprenorphine prescribing (eg, clinics may have not had X-waivered prescribers, or had policies that did not encourage use of buprenorphine outside of a specialty addiction treatment setting). Brief counseling interventions that a physician or other clinic staff member conducts following a positive screening were not extractable in this dataset. We were unable to disaggregate Latino populations by country of origin, and there may be differing patterns of alcohol and other drug use and rates of diagnosis of AUD or OUD by country of origin.^40^ We recognize that Latino and Black populations are not monolithic. Finally, we aimed to adjust for state-level heterogeneity by including Medicaid expansion status as a covariate but acknowledge that unmeasured state-level variation may remain.

## Conclusions

Among a diverse cohort of patients served by community-based primary care clinics we found overall low likelihood of screening for alcohol and drug use. Latino patients who preferred Spanish had increased odds of receiving screening for unhealthy alcohol and drug use. However, all minority populations and ethnicity with language preference groups with alcohol or opioid use disorders were less likely to have been prescribed medications for addiction treatment compared with non-Hispanic White patients. While much work has been done at the community clinic level to achieve equitable care for underserved patients these clinics serve, our findings suggest more can be done to achieve equitable substance use care in community primary care in a time of rapid increase in substance use problems in these populations across the US.

## Notes

Supplement. Data Sharing Statement
