# Supplementary material for: Race, Ethnicity, and Language Disparities in Alcohol and Drug Screening and Medication Treatment
Source: JAMA Netw Open. 2026 May 13;9(5):e2612319. doi: 10.1001/jamanetworkopen.2026.12319 (PMC13173389; doi:10.1001/jamanetworkopen.2026.12319)
Supplement: Supplement. — Data Sharing Statement [file jamanetwopen-e2612319-s001.pdf]

Data Sharing Statement

Chan. Race, Ethnicity, and Language Disparities in Alcohol and Drug Screening and Medication Treatment. *JAMA Netw Open*. Published May 13, 2026.
doi:10.1001/jamanetworkopen.2026.12319

Data
Data available: No

Additional Information
Explanation for why data not available: Individual requests for data will be considered on a case by case basis with appropriate DUA setup.
